# Novel Salvage Therapy Options for Initial Treatment of Relapsed/Refractory Classical Hodgkin’s Lymphoma: So Many Options, How to Choose?

**DOI:** 10.3390/cancers14143526

**Published:** 2022-07-20

**Authors:** Radhika Takiar, Yasmin Karimi

**Affiliations:** Division of Hematology and Medical Oncology, Department of Internal Medicine, Michigan Medicine, 1500 East Medical Center Drive, Ann Arbor, MI 48109, USA; rtakiar@med.umich.edu

**Keywords:** relapsed/refractory, classical Hodgkin lymphoma, immunotherapy, brentuximab vedotin, salvage chemotherapy, autologous stem cell transplantation, pembrolizumab, nivolumab, radiation

## Abstract

**Simple Summary:**

Relapsed/refractory classical Hodgkin’s lymphoma (cHL) accounts for 10–30% of patients. Historically, such patients were treated with salvage chemotherapy regimens followed by autologous stem cell transplantation. Introduction of novel agents such as brentuximab vedotin and immunotherapy to the treatment algorithm for cHL may change the choice of salvage therapy regimens. The purpose of this article is to review the various salvage regimens used to treat first relapse or primary refractory disease that incorporate novel agents, discuss the recent literature and propose an algorithm to determine the treatment approach for this patient population.

**Abstract:**

The treatment landscape for relapsed/refractory classical Hodgkin’s lymphoma (cHL) has evolved with the introduction of several novel agents. Historically, the standard of care for relapsed cHL was salvage chemotherapy followed by autologous stem cell transplant (ASCT). However, many patients are ineligible for ASCT or will have poor responses to salvage chemotherapy and ASCT. Brentuximab vedotin (BV) and checkpoint inhibitors (nivolumab/pembrolizumab) were initially approved in the post-ASCT setting. However, as a result of excellent responses and durable outcomes in this setting, they are now being studied and explored in earlier lines of therapy. Additionally, these agents are also being studied for post-transplant consolidation and maintenance with promising results in improving progression-free survival. We will review current salvage therapy options involving these novel agents and provide comparisons between regimens to aid the clinician in selecting the appropriate salvage regimen for patients who progress after first-line therapy.

## 1. Background

Hodgkin lymphoma (HL) accounts for 10% of all lymphomas and is predominantly classical HL (cHL) [1,2]. According to SEER data, the 5-year relative survival from 2011–2017 has been 88% [3] and is higher among those with early-stage disease [2]. The majority of patients are cured with front-line chemotherapy; however, the challenge lies among those with relapsed or refractory disease, which account for 10–30% and 5–10% of patients, respectively [4]. Those with advanced-stage disease (Stage III or IV) are more likely to relapse at a rate of 30% [5] compared with 5–10% among those with limited-stage disease [6]. Salvage chemotherapy followed by autologous stem cell transplantation (ASCT) has been the historical standard of care, though it is now either being combined or replaced with novel agents such as brentuximab vedotin (BV) and checkpoint inhibitors to attain more durable responses [7].

## 2. Salvage Chemotherapy

The standard of care for relapsed/refractory (R/R) cHL is high-dose chemotherapy followed by ASCT which was established based on two randomized trials. The British National Lymphoma group randomized relapsed patients to BEAM (carmustine, etoposide, cytarabine, melphalan) followed by ASCT or mini-BEAM alone, noting an improvement in progression-free survival (PFS) with chemotherapy followed by ASCT [8]. Similarly, the German Hodgkin’s Lymphoma Study Group randomized relapsed cHL patients to either dexamethasone-BEAM (Dexa-BEAM) or Dexa-BEAM with ASCT. Three-year freedom from treatment failure was significantly improved among patients who received BEAM-ASCT (55% vs. 34%, *p* = 0.019) [9]. To assess for chemosensitive disease, patients are given salvage therapy with a platinum-based or gemcitabine-based regimen as outlined in Table 1 [10,11,12,13,14,15,16,17,18,19]. Salvage chemotherapy has shown similar overall response rates (ORR) as depicted in Table 1, though the treatments have not been compared directly. Patients with chemosensitive disease are more likely to have improved outcomes after ASCT, with a study by Moskowitz et al. reporting a 10-year OS difference of 66% (chemosensitive) vs. 17% (chemorefractory) [20]. For this reason, optimizing complete response (CR) rates prior to ASCT has been a focus of clinical trials. With traditional salvage chemotherapy and ASCT, around 50% of patients will not be cured by this approach [21], which has led to the ongoing investigation of novel agents. The goal of incorporating novel agents as part of salvage chemotherapy is to improve CR rates pre-transplant and thus long-term outcomes after ASCT.

## 3. Brentuximab Vedotin

Nearly all cases of cHL express CD30, which led to the development of targeted therapy with BV [23]. BV is a CD30-directed antibody drug conjugated to mono-methyl auristatin molecule (MMAE), an anti-tubulin agent which leads to apoptosis [24]. The introduction of BV for R/R cHL has shown high ORR with minimal toxicity and changed the landscape of treatment as outlined in Table 2. Initially, BV was studied among patients with disease progression following ASCT. An international phase II trial evaluated BV (1.8 mg/kg every 3 weeks, maximum of 16 cycles) among R/R cHL patients after ASCT and showed ORR 75% and CR 34% in this heavily pre-treated population. Although any grade neuropathy was 42%, only 8% was grade 3 or higher and typically neuropathy was reversible with drug discontinuation or dose-reduction [25]. Longer term survival outcomes were later reported with 5-year OS and PFS rates of 41% and 22%, respectively. Among those who attained CR, the 5-year OS and PFS rates were 64% and 52%, respectively, suggesting that BV post-ASCT leads to durable disease control with minimal toxicity, especially among patients with CR [26].

Based on promising results among relapsed patients following ASCT, BV was then studied in the pre-transplant salvage setting. The first of many trials to investigate BV in the pre-transplant setting was a phase II trial in which Chen et al. treated 37 R/R patients with BV alone (4 cycles) as a second-line therapy prior to high-dose chemotherapy/ASCT, and ORR was 68% and CR 35%. The use of BV monotherapy as second-line therapy spared approximately 50% of patients from multi-agent salvage chemotherapy [27]. Furthermore, single-agent BV was also evaluated with positron-emission tomography (PET)-based approaches. A multi-center study demonstrated that BV as a second-line therapy is well tolerated and effective, though BV dose-escalation (2.4 mg/kg) based on PET did not improve responses. Similar to the aforementioned study, approximately half the patients were able to avoid salvage chemotherapy before transplant [28].

**Table 2:** Summary of Trials with BV-based Agents in Relapsed/Refractory cHL.

Regimen	Trial	Inclusion	# Pts	ORR %	CR %	mPFS or PFS	OS %	Toxicity
BV-post ASCT	Younes [25] Chen [26]	R/R after ≥1 line therapy and ASCT	102	75	34	9.3 mo	5Y: 41	Any grade neuropathy (15%) Nausea (35%)
BV-pre ASCT	Chen et al. [27]	R/R HL after 1 line therapy	37	68	35			G3 neutropenia (5%) G1 neuropathy (49%) Rash (40%)
BV → aug ICE pre ASCT	Moskowitz [29,30]	R/R after ≥1 line therapy	65	76	76	6Y: 73%	2Y: 95 6Y: 86	G1–2 neuropathy (49%)
BV → salvage pre ASCT	Herrera [28]	R/R HL after 1 line therapy	56	75	43	2Y: 67%	2Y: 93	Any grade neuropathy (63%) * Any grade rash (50%) *
BV + benda	LaCasce [31,32]	R/R HL after 1 line therapy	53	93	74	2Y: 63% 3Y: 60%	3Y: 92	Infusion reactions (56%) Any grade neuropathy (54%) G3 Neuropathy (3.6%)
BV + benda **	O’Connor [33]	R/R HL after ≥ line therapy	65	71	32			G3–4 Neutropenia (25%) G3 lung infection (14%)
BV + benda	Kalac [34]	Refractory after ≥1 line therapy + 90% prior auto	10	100	90			
BV + benda ***	Picardi [35]	R/R after ≥1 line therapy + 25% ASCT	20	100	100	2Y: 94%		G3–4 Neutropenia (15%) CMV viremia (25%)
BV + benda	Broccoli [36]	1st salvage No prior txp	40	84	79	3Y:67%	3Y: 88	G3–4 Neutropenia (27%) Any grade skin rxn (28%) G1 neuropathy (1.8%)
BV + benda − retrospective	Iannitto [37]	Refractory or 2nd relapse HL; 25% txp	47	79	49	18 mo	2Y: 72	G3–4 Neutropenia (23%) G3–4 Neuropathy (11%)
BV + ICE × 2	Cassaday [38]	1st salvage or primary refractory	23		87			G3–4 Sepsis/Neutropenic Fever (48%) Any grade neuropathy (30%) G3 neuropathy (4.3%)
BV + ICE × 2–3	Stamatoullas [39]	R/R after 1 line therapy	39		69	1Y: 69%		G3–4 Hematologic Toxicity (71%) G3–4 Infection (21%)
Dose-dense BV + ICE	Lynch [40]	R/R after 1 line therapy; no prior txp	45	91	74			G3–4 Neutropenia (73%) G3–4 Thrombocytopenia (80%) Sepsis (13%) G3 neuropathy (2%)
BV + DHAP	Hagenbeek [41] Kersten [42]	R/R after 1 line therapy	52		81	2Y: 74%	2Y: 95	G3–4 Neutropenia (65%) G3–4 Thrombocytopenia (76%) G3–4 infections (30%)
BV + ESHAP	Garcia-Sanz [43]	R/R after 1 line therapy	66	91	70			G3–4 neutropenia (32%) G3–4 thrombocytopenia (21%)
Summary of Trials with Immunotherapy-based Agents in Relapsed/Refractory cHL.								
Nivolumab	Ansell [44]	R/R after ≥1 line therapy (BV/chemo/ASCT)	23	87	17			Any grade rash (22%) Any grade thrombocytopenia (17%) Pancreatitis (4%)
Nivolumab	Younes [45]	R/R after BV and ASCT	80	66	9			G1–2 fatigue (25%) G1–2 infusion-related rxn (20%) Any grade rash (16%) Pneumonitis (3%)
Nivolumab	Armand [46]	R/R after ASCT; BV naïve, BV after ASCT, BV before/after ASCT	243	69		14.7 mo		Any grade fatigue (23%) Diarrhea (15%) G3–4 elevated lipase (5%) G3–4 neutropenia (3%)
Pembrolizumab	Chen [47,48]	R/R post-ASCT + BV, chemo-resistant w/o ASCT, ASCT-BV	210	72	28	13.7 mo		G3 Neutropenia (2.4%) G3 Diarrhea (1.4%)
BV + Nivolumab	Advani [49]	R/R after 1 line therapy (pre ASCT)	91	85	67	3Y: 91%		G4 Pneumonitis (3%) G3–4 Neutropenia (5%) Guillain Barre syndrome (1%)
BV + Nivolumab + Ipilimumab	Diefenbach [50]	R/R after ≥1 line of therapy; regardless of prior txp	61	76 (ipi) 89 (nivo) 82 (triplet)		1.2 yr (ipi)		G3–4 rash (9–26%) Grade 5 dyspnea (5%) * triplet G4 Stevens Johnson syndrome (5%) * triplet
Nivolumab → ICE	Herrera [51]	R/R after 1 line therapy (pre ASCT)	39	89	86	1Y: 79	1Y: 97	Grade 3 thrombocytopenia (3%) * Nivo alone Grade 4 altered mental status (3%) * Nivo alone Grade 3–4 neutropenia (3%) * NICE
Pembrolizumab + GVD	Moskowitz [52,53]	R/R after 1 line therapy (pre ASCT)	38	100	95			G3 elevated LFTs (10%) G3 Neutropenia (10%) G3 mucositis (5%)
Pembro vs. BV (KEYNOTE 204)	Kuruvilla [54]	R/R (post ASCT) or txp ineligible	304	66% (pembro) vs. 54% (BV)		13.2 mo (pembro) vs. 8.3 mo (BV)		G3–5 pneumonitis (1–4%) G3–5 neutropenia (2–7%)

* Among escalated BV patients; ** Included anaplastic large TCL patients (1); *** Bendamustine dose 120 mg/m^2^—higher rate of CMV viremia.

## 4. BV Combinations

Although BV monotherapy has shown response rates of around 70%, the rate of CR was suboptimal, prompting investigation of BV in combination with chemotherapy. One approach that has been studied is sequential therapy with BV and chemotherapy.

One such approach has been a PET adapted strategy. A single-center phase II trial administered BV (1.2 mg/kg weekly for 3 weeks of 28-day cycles) as second-line therapy for 2 cycles (cohort 1) or 3 cycles (cohort 2) followed by PET. Those with Deauville 1–2 were defined as PET-negative and proceeded to ASCT; however, those with Deauville 3–5 were considered PET-positive and were treated with augmented ifosfamide, carboplatin, etoposide (aug-ICE) for two cycles prior to re-evaluation for ASCT. Of 45 patients in cohort 1, 76% attained PET negativity overall with 27% PET-negative after BV therapy. All the patients who completed the treatment as per the protocol (n = 44) eventually proceeded to transplant. Of these patients, 49% experienced G1/2 neuropathy and there was no reported G3 or higher neuropathy. Two-year event-free survival (EFS) was similar among BV alone (92%) and BV with salvage augmented ICE (91%) [29]. The only risk factor predictive of inferior EFS after multi-variate analysis was the presence of PET positivity pre-transplant regardless of if patients received BV alone or BV followed by augmented ICE [55]. Upon comparison of both cohorts, an additional cycle of BV did not significantly change rates of CR. Outcomes among patients were durable with 6-year OS rate of 86% and a PFS rate of 73% [30].

Another expansion of a prior study [27] involved a PET-adapted approach for dose-escalation of second-line BV with sequential salvage combination chemotherapy for those who did not attain CR. In this study, 39% (n = 22) of patients required salvage chemotherapy (ICE, DICE, IGEV, GND). After ASCT, the 2-year PFS and OS rates were 67% and 93%, respectively, and 49% of patients experienced G1 neuropathy with no G2 or higher neuropathy reported. Interestingly, the 2-year PFS rate for BV alone was 77% vs. 57% for BV with chemotherapy, though not statistically significant. This raises the ongoing question of whether non-chemotherapy based salvage regimens are sufficient and/or superior [28].

In addition to PET adapted approaches, BV has been studied in combination with chemotherapy in the salvage setting. A larger multi-center phase I/II trial by LaCasce et al. evaluated BV (1.8 mg/kg on day 1) with bendamustine (90 mg/m^2^ on days 1 and 2 of 21-day cycles) for 2–6 cycles followed by optional ASCT and BV maintenance [32]. Of all patients (n = 53), 74% attained CR, of which 87% attained CR after two cycles alone. Approximately 75% of patients (n = 40), proceeded to transplant. The ORR was 93% with an estimated 2-year PFS rate of 63% overall and 70% among the ASCT group. The most prevalent adverse event was infusion related reactions (IRRs), which were seen in 56% of patients and required an amendment in the trial requiring incorporation of pre-medications. This lowered the severity of IRRs but did not significantly change the incidence. Long-term follow-up reported a 3-year PFS rate of 60% overall and a 3-year OS rate of 92%, which was not significantly different among transplanted versus non-transplanted patients. Any grade neuropathy was experienced by 54% of patients but only 3.6% experienced G3 neuropathy. Additionally, 63% of reported cases of peripheral neuropathy had resolved or improved [31]. Other trials or retrospective data with BV and bendamustine report ORR ranging from 71 to 100% as outlined in Table 2 [33,34,35,36,37].

The addition of BV concurrently to combination salvage chemotherapy has also been explored. A phase I/II trial among patients with first relapse or primary refractory cHL involved BV (either at 1.2 mg/kg or 1.5 mg/kg) with concurrent ICE for two cycles. Of 23 patients, 87% attained complete metabolic response (CMR) by PET imaging and among those who attained CMR, 86% underwent ASCT (19 of 22 patients) [38]. A similar trial by Stamatoullas et al. combined BV (1.8 mg/kg) with ICE for 2–3 cycles and reported a CMR of 69% [39]. To better understand the optimal dosing of BV with ICE in the second-line setting, a phase I/II study was performed at the University of Washington. They utilized a 3 + 3 dose escalation design and established the maximum tolerated dose to be BV at 1.5 mg/kg (max 150 mg) on days 1 and 8 with standard ICE. The majority of patients completed two cycles of therapy (91%), though there was one treatment related-death from multi-organ failure. The ORR was 91% with 74% attaining CR (below study target CR: 78%) and 86% of patients proceeding with ASCT. Weekly dose-dense administration was feasible and attained excellent response rates, though it was associated with high rates of grade 3/4 neutropenia and thrombocytopenia at 73% and 80%, respectively [40].

The combination of BV with dexamethasone, cisplatin, and cytarabine (DHAP) has also been explored for second-line salvage therapy, and results from 2021 demonstrated that 92% of patients completed three cycles of therapy with a CMR rate of 81% and a majority of patients (85%) proceeded to ASCT. After a median follow-up of 27 months, the 2-year PFS rate was 74% and the 2-year OS rate was 95% [41,42]. The Spanish Lymphoma Group and Bone Marrow Transplantation (GELTAMO) combined a regimen they use commonly: etoposide, methylprednisolone, cytarabine and cisplatin (ESHAP) with BV in a phase I/II trial. After confirming safety, they utilized BV at 1.8 mg/kg with ESHAP for three cycles followed by a single dose of BV. The outcomes were similar to other combinations with an ORR of 91% and a CR of 70%. Additionally, 91% of patients successfully underwent ASCT without engraftment failure [43]. Therefore, several concurrent combinations of BV with chemotherapy exist that increase the likelihood of attaining CR pre-ASCT and could lead to improved long-term outcomes. These regimens have yielded excellent ORR and allowed patients to proceed to ASCT; however, at the expense of additional toxicities.

Although combinations of BV with salvage chemotherapy yielded improved CR rates ranging from 70 to 80%, as outlined in Table 2, they also came with increased hematologic and non-hematologic toxicities. For example, the rates of grade 3/4 neutropenia and thrombocytopenia were 65% and 76%, respectively, among patients receiving BV-DHAP in the BRaVE study [42]. Similarly, rates of grade 3/4 neutropenia and thrombocytopenia were 47% and 50%, respectively while patients were receiving BV-ESHAP [43]. Febrile neutropenia occurred at a rate of 25–35% among patients on either of these regimens [41,42,43]. Non-hematologic grade 3–4 toxicities seen during BV-DHAP included elevated liver enzymes (18%) and electrolyte disorders (10%) [42]. Thus, despite excellent response rates, BV with chemotherapy may be a less preferred option among those who may incur more longstanding consequences due to the toxicities of such regimens.

## 5. Immunotherapy

Although BV has changed the landscape for R/R cHL, some patients may not respond to BV-based therapy or have been previously treated with BV as part of a first-line therapy, which is what prompted investigation of other agents, specifically immunotherapy.

Incorporation of immunotherapy for cHL was explored based on pre-clinical data noting relevance of the programmed cell death-1 (PD-1) pathway within this disease. A genetic abnormality among HL cases are chromosomal 9p24.1 amplification and alterations. Preclinical studies have demonstrated that among 9p24.1 amplified cHL cells, there is an increased expression of PD-1 ligand genes, specifically programmed cell death-1 ligand (PD-L1) and PD-L2 [56]. Additionally, this amplification increases Janus 2 kinase (JAK2) activity which further induces increased PD-L1 and PD-L2 expression on Reed-Sternberg (RS) cells [56]. Activation of the PD-1 pathway by PD-L1 and PD-L2 engagement decreases T-cell mediated immune responses [57]. Thus, it was postulated that RS cells with high PD-L1 and PD-L2 expression are evading immune detection, which led to investigation of PD-1 checkpoint inhibitors, specifically nivolumab and pembrolizumab, for treatment of cHL.

Initial studies explored the safety and efficacy of nivolumab among R/R HL. Nivolumab was evaluated for heavily pre-treated R/R cHL patients in a phase I trial, most of whom had received ≥3 lines of therapy and ASCT. The treatment consisted of nivolumab at 3 mg/kg at week 1, week 4 and then every 2 weeks until progression or CR for a maximum of 2 years. Of 23 patients, the ORR was 87% with a CR at 17%, and toxicity was limited to grade 1 or 2 [44]. Similarly, Younes et al. performed a multi-center phase 2 study, CheckMate 205, evaluating the efficacy of nivolumab at 3 mg/kg every 2 weeks among patients who had failed several lines of therapy (range 3–15), including BV and ASCT. The response rate assessment varied with an ORR of 66% vs. 78% and a CR of 9% vs. 28% reported by the independent radiologic review committee and investigator review, respectively. The median duration of response (DOR) was 7.8 months. Of 80 patients, only one patient discontinued therapy from autoimmune hepatitis and two patients had pneumonitis which responded to steroids [45]. Several retrospective reviews of previously treated R/R cHL patients who were given nivolumab have cited similar response rates: ORR 60–70% and CR 20–40% [58,59,60].

As previously mentioned, CheckMate 205 was a phase II trial that evaluated nivolumab at 3 mg/kg given every 2 weeks until disease progression or unacceptable toxicity among those with R/R HL after failure of ASCT. Three cohorts were assessed: BV-naïve, BV given after ASCT and BV given before and/or after ASCT. After a median follow-up of 18 months, the ORR was 69% (CR 16%) across all cohorts with the median DOR lasting 16.6 months and a median PFS of 14.7 months. Of note is that 44 patients (18%) proceeded to allogeneic stem cell transplantation due to progressive disease [46]. The updated results presented at the International Conference on Malignant Lymphoma in 2021, demonstrated ongoing durable responses with the ORR at 71%, CR at 21%, a median DOR of 18 months and a 5-year OS of 71% and with a median follow-up of 58 months [61].

Similarly, pembrolizumab was also studied among R/R HL patients. In the phase II trial, KEYNOTE-087 [47], patients were stratified into three cohorts: post ASCT and subsequent BV, post-salvage chemotherapy and BV (chemo-resistant disease), and those post-ASCT without subsequent BV. Pembrolizumab 200 mg was given every 3 weeks for 2 years or until disease progression or unacceptable toxicity. The two-year follow up results reported an ORR of 72%, a CR of 28%, a median DOR of 16.5 months and a median PFS rate of 13.7 months in all cohorts. Those who attained CR had higher median DOR compared to partial responders; however, 64% of patients were on therapy for at least 1 year before achieving CR. Regardless of the initial therapy, the use of pembrolizumab led to durable and deep responses with minimal grade 3 or 4 adverse events [48]. The five-year follow-up of KEYNOTE-087 demonstrated similar ongoing responses: ORR, 71%; CR, 27.6%; and 5-year OS, 70.7% [62]. Overall, immune checkpoint inhibitors are well tolerated, though have led to treatment discontinuation in 5–8% of patients due to immune-related adverse events (IrAE) [46,48].

Although CR rates appear lower in these immunotherapy-based trials [46,47,48], the PFS rate is similar to that of BV-based regimens, which raises the question of whether PET is truly capturing CR. Pseudo-progression, a radiologic phenomenon seen with use of immunotherapy, is the appearance of increasing or new lesions due to therapeutic immune activation rather than true progressive disease. To address this issue, an additional category of “indeterminate responses (IR)” was created in 2016, now known as lymphoma response to immunomodulatory therapy criteria (LYRIC) [63]. The incorporation of LYRIC to response assessments for immunotherapy-based regimens may be more accurate, though has not been well-studied prospectively. Despite lower response rates seen with immunotherapy, median DOR, PFS and OS suggest excellent disease control.

Although the above data are single-arm phase I/II studies without comparative arms, the high response rates are promising and have led to the approval of these regimens in the NCCN guidelines [64]. Currently, the FDA has approved pembrolizumab and BV as second-line agents following relapse with nivolumab being approved as third-line. The European Medicines Agency (EMA) has approved pembrolizumab or BV among R/R cHL patients following ASCT or transplant ineligible patients who failed ≥ 2 prior lines of therapy. They have approved nivolumab for R/R cHL after ASCT and when used in conjunction with BV.

Other anti PD-1 therapies, such as sintilimab, tiselizumab, and camrelizumab, have mostly been studied in phase 2 trials in exclusively Chinese patients with R/R cHL. Sintilimab was administered to patients (200 mg every 3 weeks) with R/R cHL after ≥2 prior lines of therapy until progressive disease, death or unacceptable toxicity. After a median follow-up of 10.5 months, the independent radiological review committee (IRCC) reported the ORR was 80.4% with 34% attaining CR [65]. Tislelizumab was studied in R/R cHL patients with a median of three prior lines of therapy and had similar response rates: ORR, 87.1%; and CR, 62.9% [66]. Camrelizumab was given at 200 mg every 2 weeks and after a median follow-up of 12.9 months, the ORR was 76% with 28% achieving CR [67]. These PD-1 inhibitors are not currently approved or available for R/R cHL patients outside of China.

## 6. BV and Immunotherapy Combinations

Due to promising responses as single-agents with minimal toxicities, BV and immunotherapy combinations have also been studied in the salvage setting. A recent phase I/II trial reports 3-year outcomes with the combination of BV-nivolumab as a second-line therapy for primary refractory or relapsed cHL [49]. Patients received BV-nivolumab either as staggered dosing with BV (1.8 mg/kg) on day 1 and nivolumab (3 mg/kg) on day 8 for cycle 1 followed by both on day 1 for cycles 2–4, every 3 weeks or as same day dosing for all 4 cycles. This led to an ORR of 85%, a CR of 67% and an estimated 3-year PFS rate of 77% for all patients. Following four cycles of BV-nivolumab, 67 patients (74%) proceeded to ASCT, and among this cohort, the 3-year PFS was excellent at 91%. As seen in other trials, the 3-year PFS was significantly worse among primary refractory patients at 61% versus 90% for relapsed disease. Overall, this combination was well tolerated despite infusion-related reactions (43%). The prevalence of IrAE requiring systemic corticosteroids was 18%, most commonly pneumonitis or rash, though none led to therapy discontinuation.

Furthermore, to expand on the combination of BV-nivolumab, ongoing trials are investigating the benefit of the addition of CTLA4 agents, such as ipilimumab. A phase I/II trial compared BV-ipilimumab, BV-nivolumab and BV with both nivolumab and ipilimumab (triplet therapy) to establish safety and efficacy [50]. Although the ORR among all three groups ranged from 76 to 89%, there were significant toxicities among each cohort, but notably worse with the triplet therapy. Grade 3–4 treatment related adverse events were seen in 43%, 16% and 50% of the ipilimumab group, nivolumab group and triplet groups, respectively, with maculopapular rash accounting for the majority of cases. There were two treatment-related deaths from pneumonitis, and one of the most common reasons for treatment discontinuation was treatment-related adverse events, accounting for 32% in the triplet group. Although this is an ongoing phase II study, based on preliminary data, the toxicities related to triplet therapy will need to be heavily considered prior to implementation [50].

## 7. Immunotherapy and Chemotherapy Combinations

Several trials are investigating the efficacy of immunotherapy as a salvage treatment without BV, by combining it with standard salvage chemotherapy regimens. For example, a sequential, PET-adapted trial of nivolumab-ICE (NICE) administered nivolumab at 3 mg/kg every 2 weeks for up to 6 cycles, and those with CR after cycle 6 proceeded to ASCT, whereas those without CR were given two cycles of NICE [51]. Upon completion of nivolumab alone, 26 patients (70%) attained CR, and of those who received NICE, 6 patients (86%) attained CR. Of the 37 patients, the majority (89%) proceeded with a transplant, either after nivolumab alone or NICE. Overall, the regimens were well tolerated with limited grade 3–4 toxicities (one patient with thrombocytopenia and one patient with altered mental status and tumor lysis syndrome) [51].

Another combination of second-line therapy involving a salvage chemotherapy regimen with the addition of immunotherapy is pembrolizumab with gemcitabine, vinorelbine, and liposomal doxorubicin (GVD). A phase II trial evaluated use of pembro-GVD given for 2–4 cycles in transplant eligible patients as a second-line therapy; those who attained CR following 2–4 cycles proceeded to ASCT [52,53]. The majority of patients (31/39) only required two cycles of pembro-GVD. Of the 38 patients, the ORR was 100% with a CR 95%. Of 36 transplanted patients, all remained in remission at a median follow-up of 13.5 months. Although IrAEs were prevalent with any-grade rash (49%), hyperthyroidism (13%) and transaminitis (41%); the grade 3 or higher toxicities were minimal with transaminitis and neutropenia each accounting for 10%. The results from this trial are promising, especially considering that 41% of patients had primary refractory disease and 38% had relapsed within 1 year upon enrollment, though longer follow-up data will be necessary to assess whether the responses remain durable.

Although the advent of novel therapies such as BV and immunotherapy for treatment of R/R cHL show promising results, the optimal sequencing for second-line therapy remains unclear in the absence of comparative trials in this setting.

## 8. Transplant Ineligible

Historically, outcomes among transplant ineligible and elderly patients (≥60 years) have been poor with median OS ranging from 1 to 2 years [68], though, incorporation of novel agents such as BV and immunotherapy are improving the outlook for such patients. The goal of treatment for this patient population is to achieve and maintain disease control.

Single agent BV or combinations, such as BV-bendamustine, still have activity among non-transplanted patients. LaCasce et al. reported outcomes of R/R patients who were treated with BV-bendamustine followed by optional ASCT and BV maintenance [32]. Although the majority of patients underwent ASCT (75%), the non-transplanted cohort still had an excellent ORR (85%) and similar durations of CR. At a median follow up of 23.0 months, the 2-year OS and PFS rates were 94.2% and 62.6%, respectively, in the overall population, including patients who did not undergo ASCT. Side effects were discussed earlier, but among the combination arm, these were primarily infusion related reactions (56%), grade 3/4 lymphopenia (11%), grade 3/4 rash (9%), grade 3/4 hypotension (7%) and any grade peripheral neuropathy (24%). Overall, this regimen was well tolerated, which makes it a promising option among patients who are transplant ineligible for reasons such as age and comorbidities.

A recent phase III international study, KEYNOTE-204, evaluated outcomes of pembrolizumab monotherapy compared with BV among patients who relapsed following ASCT or those who were transplant ineligible [54]. Patients were randomized to receiving either pembrolizumab at 200 mg every 3 weeks or BV at 1.8 mg/kg every 3 weeks for a maximum of 35 cycles or until progressive disease, unacceptable toxicity or investigator decision. Although this study allowed patients to have prior exposure to BV, this number was small (5%). Of 304 patients, 63% were ineligible for ASCT primarily due to chemo refractory disease (44%), but also due to age (8%), comorbidities (2%), or other factors (9%), such as patient refusal, social reasons and physician choice. At the median follow-up at approximately 26 months, the median PFS was higher among the pembrolizumab group (13.2 months vs. 8.3 months, *p* = 0.0027). A subgroup analysis demonstrated that pembrolizumab had more favorable PFS rates among those with primary refractory disease and among patients who did not have prior ASCT. Although the ORR was not statistically different among the two groups, pembrolizumab led to longer median DOR (20.7 months vs. 13.8 months). IrAEs were more common among those treated with pembrolizumab (33% vs. 7%); however, grade 3–5 events only accounted for 7% in the pembrolizumab group.

The incorporation of immunotherapy or BV may become a preferred approach among patients who are transplant ineligible, especially since these agents are better tolerated than cytotoxic chemotherapy regimens and have led to improved and durable response rates.

## 9. Post-Transplant Maintenance/Consolidation

Prior data has shown that patients who relapse following ASCT have poor outcomes, especially if relapse occurs within the first year post-ASCT as median survival is approximately 1 year [69]. The AETHERA trial sought to investigate whether incorporation of BV as consolidation following high dose chemotherapy and ASCT could improve outcomes among those at high risk of relapse or disease progression [70,71]. Patients were defined as high-risk if they had primary refractory disease, relapse <12 months after frontline therapy or extranodal relapse. This was a phase III international trial that randomized BV-naïve patients to BV at 1.8 mg/kg every 3 weeks or a placebo for up to 16 cycles starting at 30–45 days following ASCT. At the 5-year follow up, the 5-year PFS was 59% and 41% with BV and placebo, respectively (HR, 0.521; 95% CI, 0.379–0.717). The benefit of BV consolidation was most pronounced among patients with two of the aforementioned high-risk features. In a post hoc analysis of the AETHERA trial, the PFS benefit was more pronounced in patients with ≥2 (n = 280) or ≥3 (n = 166) of five risk factors, namely relapse < 12 months or refractoriness to front-line therapy, partial response (PR) or stable disease (SD) as best response to most recent salvage therapy, extranodal disease at pre-ASCT relapse, B symptoms at pre-ASCT relapse or ≥2 prior salvage therapies. To date, this has not translated to improved overall survival and the use of BV was associated with higher incidence of toxicity, specifically peripheral neuropathy (67%) and neutropenia (35%). Additionally, BV consolidation reduced need for subsequent therapy or allogeneic SCT compared with the placebo. The role of post-transplant consolidation with BV is becoming unclear since BV can be incorporated into front-line therapy based on the ECHELON-1 trial [72], and the AETHERA trial was strictly analyzing BV-naïve patients [70,71]. In practice, BV consolidation is often used when patients have used BV as part of their salvage regimen. This is commonly done by completion of 16 cycles of BV accounting for therapy received prior to transplant. Clinically, it is not uncommon for dose reductions or therapy cessation prior to completion of 16 cycles due to increasing cumulative neuropathy.

Additionally, there is an ongoing investigation analyzing the use of immunotherapy as a component of post-transplant consolidation. A phase II trial hypothesized that use of pembrolizumab post-ASCT among high-risk R/R cHL patients would improve PFS (at 18 months post ASCT) from 60 to 80% [73]. The patients were given pembrolizumab at 200 mg every 3 weeks for up to eight cycles; of 30 patients, 77% completed all eight cycles. The 18-month PFS rate was 82% and the 18-month OS rate was 100%. Although the rate of IrAEs was 43%, there were limited grade 3 or higher IrAE.

Studies have also investigated combining BV and immunotherapy for consolidation post-ASCT. A phase II trial [74] enrolled patients with R/R high-risk cHL, defined as primary refractory HL, relapse <1 year after completing initial therapy, extranodal disease at relapse, B symptoms at relapse or >1 salvage therapy required prior to ASCT. The patients were allowed to enroll if they had prior BV or nivolumab as long as they were not refractory to these therapies. The treatment consisted of eight cycles of BV/Nivolumab starting between 30 and 75 days post-ASCT. Fifty-nine patients were enrolled and half were able to complete all eight cycles of therapy. Equal numbers of patients had early discontinuation of BV and nivolumab (14% and 12%, respectively) with only 10% of patients discontinuing both drugs. The most common toxicities were peripheral neuropathy (51%, only 3% Grade 3), neutropenia (42%), with IrAEs requiring corticosteroids occurring in 31% of patients. The rate of IrAEs requiring corticosteroids appears to be higher than for pre ASCT patients. At a median follow-up time of 15.7 months, the 18-month PFS and OS rates were 95% and 98%, respectively. Notably, six patients in the study were not in CR at baseline after ASCT and five patients converted to CR with the study treatment and one patient remained in PR without progressive disease. Further long-term data with a larger cohort will be necessary to determine whether post-transplant consolidation with immunotherapy improves outcomes without increasing toxicities [74].

Another post-transplant maintenance strategy that is being investigated involves nivolumab among patients with high risk of relapse or progression (defined as refractory disease, relapse <12 months or relapse ≥12 months with extranodal disease after front-line therapy) [75]. Patients were given nivolumab at 240 mg every 2 weeks starting 45–180 days post-transplant for a maximum of 6 months. At data cutoff, 76% of patients had discontinued nivolumab, which was due in the majority (60%) of these cases to completion of 6 months of therapy. Due to the short median follow up, the median PFS and OS have not been reached; however, the 6-month PFS rate was 92.1% and the 12-month OS rate was 100%.

## 10. Salvage Radiation

Another therapeutic modality for the treatment of R/R cHL is radiotherapy (RT). The two scenarios for the use of radiation therapy in the treatment of initial relapse or refractory disease are (1) as consolidation prior to or following autologous stem cell transplant or (2) radiation alone for limited stage relapse or in a palliative setting [76,77,78]. Given the risk of recurrence after ASCT is highest at sites of initial involvement, clinicians have used local radiation therapy at sites of initial disease either prior to or after ASCT. There are many retrospective studies that have shown conflicting results regarding the utility of RT peri transplant [79,80,81,82,83,84,85,86,87]. There is mixed data on the role of RT following ASCT in the PET era. One retrospective study conducted in the PET era has shown a PFS benefit to the addition of RT after autologous SCT in patients with initial bulky disease or persistent disease after salvage chemotherapy and prior to autoSCT [88]. Notably, there was no difference in overall survival between patients who received RT and those not treated with RT peri transplant. However, a separate retrospective study found a trend towards higher 3-year OS and PFS rates for limited stage relapses treated with RT compared with CT alone, though this was not statistically significant [86]. Unfortunately, there are no prospective studies to guide management and decisions should be made based on clinical factors and the response to salvage treatment and ASCT. An analysis of second-line RT alone without systemic chemotherapy was performed on the German Hodgkin Study Group (GHSG) database [89]. At first treatment failure, salvage RT was given to 100 patients with 5-year freedom from treatment failure being 28% and 5-year OS at 51%. In this patient population, the majority of relapses (88%) were limited stage (Ann Arbor Stage I-II). Thus, salvage RT is an option for patients with limited-stage late relapses; however, it should be reserved for patients treated with palliative intent therapy given the high rates of progression when used without systemic therapy.

Salvage RT has also been explored in combination with systemic therapy. A combination of RT with BV [90] or immunotherapy has led to CR among R/R cHL patients, though in small case series or retrospective studies [91,92,93].

## 11. Discussion

The advent of novel therapies such as BV and immunotherapy for treatment of cHL is promising, but has created the new challenge of how to best integrate these therapies into clinical practice.

There have been some recent retrospective studies that have tried to evaluate the comparative effectiveness of various salvage regimens. A large multi-institution retrospective study compared response rates and outcomes among various salvage regimens for R/R cHL patients who underwent ASCT. This data was presented at the American Society of Hematology (ASH) Annual Meeting in 2021 [94]. The combination of BV-bendamustine had a higher ORR (92% vs. 79%, *p* < 0.001) and CR rates (80% vs. 49%, *p* < 0.001) compared with platinum-based chemotherapy. Among patients who attained CR pre-ASCT, BV-nivolumab led to higher PFS (HR 0.1, *p* < 0.05) but not OS compared with platinum-based chemotherapy. Additionally, this data further supported prior evidence that patients with PR or progressive disease had worse 2-year PFS and OS compared with those who attained pre-ASCT CR.

To further compare outcomes among BV-based vs. chemotherapy-based salvage regimens, Driessen et al. collected data from several prospective trials and matched cohorts by propensity score matching [95]. They presented their findings at the 2021 ASH meeting where a total of seven BV-chemotherapy and two chemotherapy-based trials were included with 205 patients among each matched cohort. Although the 3-year PFS was not different between the two cohorts, 3-year OS was higher in the BV cohort, though this benefit was likely due to advances in novel therapies over time that were not available during the era of the chemotherapy trials. Patients with relapsed disease had a higher PFS rate in the BV cohort though this benefit was not observed for primary refractory disease.

There is ongoing investigation assessing whether pathologic correlates may be helpful in guiding therapeutic decisions. As previously discussed, most HL cells have amplification of 9p24.1, which up-regulates PD-L1 and PD-L2 [56]. A phase II trial with nivolumab in a heavily pre-treated population reported exploratory analyses focusing on PD-L1 and PD-L2 [45]. Of 45 patients with available tumor biopsy samples, copy gain of PD-L1 and PD-L2 was present in 58%, polysomy 9 in 16% and PD-L1 and PD-L2 amplification in 27%. The PD-L1 H score represented the percentage of malignant cells with positive staining (for PD-L1 expression) multiplied by the average intensity of positive staining. Post hoc analyses found that patients with PD-L1 H scores in the higher quartiles achieved CR as opposed to those in the first quartile who had progressive disease (*p* = 0.013). Although this trend seems promising, it was a limited sample size and needs further investigation. In contrast, a recent oral abstract publication at the 2021 ASH meeting of correlative studies of a front-line trial of pembrolizumab in combination with AVD [96] showed no correlation between the PD1 pathway and responses to single agent pembrolizumab. In this study, untreated patients diagnosed with cHL were treated with three cycles of single agent pembrolizumab followed by PET/CT prior to the start of AVD therapy. After this treatment, 63.4% of patients had a complete or near complete response to single agent pembrolizumab and responses were seen even in patients with low levels of PD1 expression.

In regards to circulating tumor DNA (ctDNA), retrospective analyses have identified mutations such as XPO1 E571K that are present in the tumor and plasma and could become a useful genetic biomarker at diagnosis and for minimal residual disease (MRD) detection [97]. Prospectively, ctDNA was assessed at diagnosis and after two cycles of front-line chemotherapy for HL patients [98]. Several mutations were found at diagnosis, though became undetectable following two cycles of therapy. Whether this trend correlates with outcomes remains unclear. The use of ctDNA may eventually inform us of how “much” treatment is warranted, though at this point, it does not offer much guidance in regard to “which” next-line treatment is optimal.

**Figure 1:**
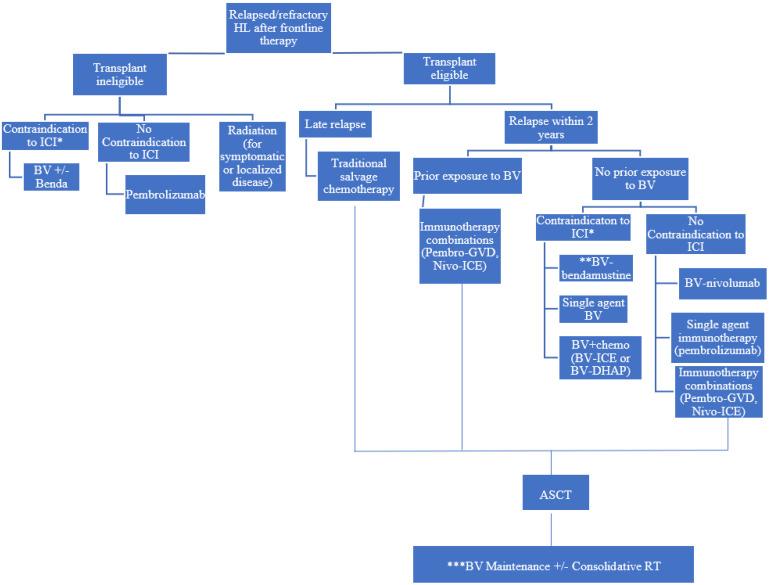
Author’s suggested approach to Selection of Initial Salvage Regimen for Relapsed/Refractory cHL. * Contraindications to immune check point inhibitors most often due to underlying autoimmune disease; ** Preferred in elderly or those who have barriers to transportation for frequent visits, *** Consider BV maintenance for high-risk patients.

In summary, the introduction of novel agents, such as BV, into front-line therapy [72] has complicated the approach to salvage therapy. Our approach to salvage therapy is outlined in Figure 1. An initial approach would be to determine whether patients are transplant eligible or ineligible. For patients who are transplant eligible, we first stratify the need to incorporate novel agents based on the duration of the first remission. Among transplant eligible patients, one approach to determine front-line salvage therapy would be stratifying according to early vs. late relapse. People who relapse early are more likely to be chemo refractory. The study conducted by Moskowitz et al. using ICE salvage chemotherapy noted a statistically improved EFS among patients who relapsed after 1 year compared with those who had relapses within a year [13]. Similarly, a study with salvage DHAP reported that early relapse (within 3–12 months) was a poor prognostic risk factor and contributed to worse PFS [12]. The AETHERA trial also considered relapse within 12 months as a high-risk feature [71]. Based on the aforementioned data, we defined early relapse as relapse occurring within 12 months of therapy.

In those who relapse late after initial therapy, it would be reasonable to treat with salvage chemotherapy alone without novel agents. However, in those who relapse within the first 12 months of initial therapy, we next assess their BV exposure. Among those who were exposed to BV as front-line or consolidative treatment, the options would be aimed at avoiding additional BV. Among those with early relapse (<12 months) or primary refractory disease, immunotherapy combinations with chemotherapy are an option or single agent pembrolizumab is possible for transplant ineligible patients, based on KEYNOTE-204 [54]. In those patients who have not received prior BV, BV-based combinations would all be appropriate, and we favor the use of BV with chemotherapy as it allows for the assessment of chemosensitivity, which predicts for improved outcomes after ASCT [20]. Due to the fact that cross-trial comparisons are challenging, selection of a regimen should be individualized to each patient based on their functional status, underlying comorbidities and preference.

Although there may not be a certain “correct answer” for which salvage therapy option is best, we must consider factors such as age, transportation barriers, location of treatment and patient preference. For the elderly cohort of cHL patients, it may be preferable to avoid cytotoxic chemotherapy due to potential toxicities; therefore, using novel agents such as immunotherapy or BV-bendamustine may be preferable. Another practical consideration that may dictate therapy is ease of transportation and location of treatment. For example, typically regimens such as ICE/DHAP require inpatient admission while others such as BV-bendamustine, pembro-GVD or immunotherapy monotherapy can be administered outpatient. Salvage radiation can also be incorporated for localized relapses or for palliative purposes. Among transplant eligible patients, salvage radiation may have a role in cytoreduction prior to ASCT or for localized relapses following ASCT [64]. A large retrospective study noted improved 2-year PFS (67% vs. 42%, *p* < 0.01) among post-transplant patients who were treated with consolidative RT for localized relapses compared with the control group [88]. Lastly, underlying comorbidities or conditions may also impact therapy decisions. For example, we would tend to avoid BV among those with baseline grade 2–3 neuropathy.

## 12. Conclusions

Although the introduction of novel agents into the treatment landscape for cHL shows promise, it also creates uncertainty when determining which salvage regimen is most appropriate. As outlined above, it may be best to individualize therapy plans based on patients’ comorbidities, preferences, social barriers, and prior therapies. Ultimately, more randomized control trials, such as KEYNOTE-204 [54], are needed to help us understand the optimal timing and combinations of immunotherapy and BV. There is ongoing investigation of ct-DNA [98] and PD-L1 [45] scores to better understand whether these can be used as predictive or prognostic markers. Outcomes for R/R HL have improved with the advent of novel agents such as BV and immunotherapy and hopefully will continue to improve as we better learn the optimal combination regimens that create long, durable responses while limiting toxicity.

## Figures and Tables

**Table 1:** Conventional Salvage Chemotherapy Regimens for Relapsed/Refractory cHL; Adapted from Vassilakopoulos et al. [22].

	Regimen	Trial	# Pts	ORR (%)	CR (%)
ESHAP	Etoposide, cytarabine, cisplatin, methylprednisolone	Aparicio [10]	22	73	41
ASHAP	Adriamycin, solumedrol, high-dose cytarabine, cisplatin	Rodriguez [11]	56	70	34
DHAP	Dexamethasone, cytarabine, cisplatin	Josting [12]	281	NR	72
ICE	Ifosfamide, carboplatin, etoposide	Moskowitz [13]	65	88	26
ICE	Ifosfamide, carboplatin, etoposide	Hertzberg [14]	6	100	67
IVOx	Ifosfamide, etoposide, oxaliplatin	Sibon [15]	34	76	32
GDP	Gemcitabine, dexamethasone, cisplatin	Baetz [16]	23	69	17
GEM-P	Gemcitabine, cisplatin, methylprednisolone	Chau [17]	21	80	24
IGEV	Ifosfamide, gemcitabine, vinorelbine, prednisone	Santoro [18]	91	81	54
BeGEV	Bendamustine, gemcitabine, vinorelbine	Santoro [19]	59		75
